# Recent progress in the role of grain boundaries in two-dimensional transition metal dichalcogenides studied using scanning tunneling microscopy/spectroscopy

**DOI:** 10.1186/s42649-023-00088-3

**Published:** 2023-07-17

**Authors:** Hyo Won Kim

**Affiliations:** Samsung Advanced Institute of Technology, Suwon, 13595 Korea

**Keywords:** Transition metal dichalcogenides, Grain boundary, Scanning tunneling microscopy, Scanning tunneling spectroscopy, Nonsymmorphic symmetry

## Abstract

Grain boundaries (GBs) are one- or two-dimensional (2D) defects, which are universal in crystals and play a crucial role in determining their mechanical, electrical, optical, and thermoelectric properties. In general, GBs tend to decrease electrical or thermal conductivity, and consequently degrade the performance of devices. However, the unusual characteristics of GBs have led to the production of a new class of memristors with 2D semiconducting transition metal dichalcogenides (TMDs) and the creation of conducting channels in 2D topological insulators. Therefore, understanding the nature of GBs and their influence on device applications emphasizes the importance of GB engineering for future 2D TMD-based devices. This review discusses recent progress made in the investigation of various roles of GBs in 2D TMDs characterized via scanning tunneling microscopy/spectroscopy.

## Introduction

The discovery of layered 2D TMDs, which consist of vertically stacked layers kept together by interlayer van der Waals interactions, has promoted extensive research on the development of next-generation devices with outstanding performance (Chhowalla et al. 2013; Manzeli et al. 2017; Yin et al. 2021). In particular, layered TMDs with several crystal polymorphs, such as hexagonal (2H), trigonal (1T), distorted trigonal (1T’), and rhombohedral (3R) phases, exhibit various electronic properties depending on their stacking sequences (semiconducting and metallic properties including low temperature phenomena such as superconductivity, charge density wave, Mott-insulator transition). Despite the additional characteristics of confinement effects, even monolayer or few-layer forms can nearly preserve the properties derived from structural configurations; this offers opportunities for their versatile applications in scaled-down devices.

GBs are inevitable in TMDs and significantly affect their mechanical, optical, electrical, and thermoelectric properties. For example, although the mechanical strength of 2D single-crystalline 2H-MoS_2_ is comparable to that of stainless steel (Wu et al. 2018), GBs can degrade such a high strength as well (Man et al. 2021). The carrier mobility in 2H-MoS_2_ can be decreased by additional carrier scattering owing to the mid-gap boundary states produced by GBs with 7–5 and 8–4–4 membered rings, whereas their mirror twin boundaries slightly increase the measured in-plane electrical conductivity (Najmaei et al. 2013; Zhou et al. 2013; Zande et al. 2013; Yu et al. 2017). A tilted boundary in 2H-MoS_2 _causes strong photoluminescence enhancement (Zande et al. 2013). Thermal transport characteristics of GBs with 7–5 membered rings in 2H-MoS_2_ are considerably altered with “U”shaped thermal conductance as the misorientation angle varies from ~ 5.06–52.26° (K Xu et al. 2022).

**Fig. 1:**
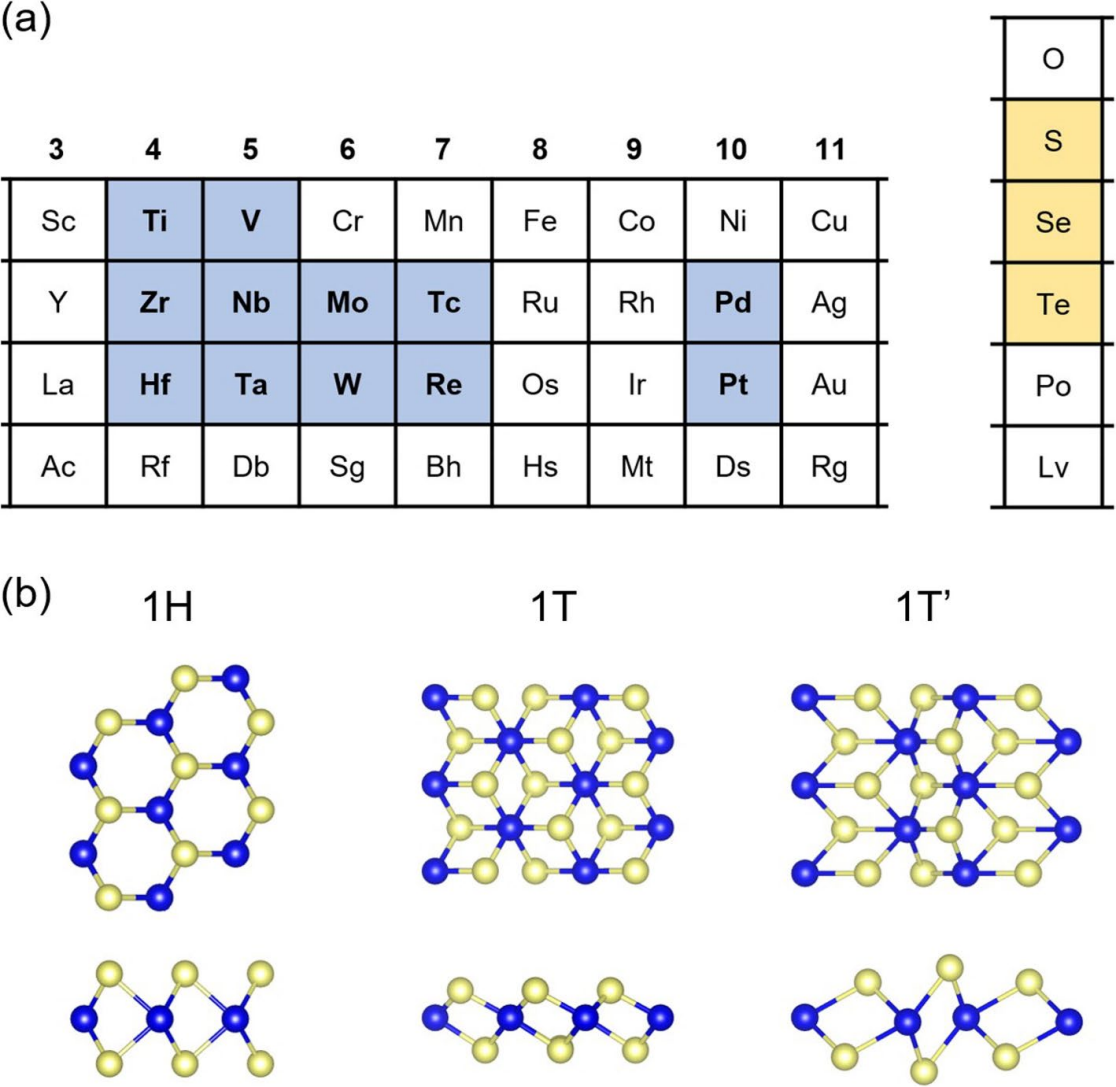
**a** Positions of transition metals (blue) and chalcogens (yellow) in the periodic table. **b** Top and side views of monolayer TMD with 1H, 1 T, and 1 T’ phases. Blue and yellow spheres represent the transition metals and chalcogens, respectively

Layered TMDs have the chemical formula of MX_2_, where M is a transition metal belonging to groups 4–10 and X represents a chalcogen (Fig. 1a). This review focuses on Group 6 TMDs, that is, M = (Mo, W) and X = (S, Se, Te). Layered Group 6 TMDs with several polymorphs such as 2H, 1T, 1T′, Td, and 3R phases have attracted much interest owing to their intriguing electronic properties, ranging from semiconducting to 2D quantum spin Hall insulator, Weyl semimetallic, and superconducting regimes. Hexagonal group 6 TMDs have a proper bandgap (1–2 eV) for optical and electronic device applications, and they can be reversibly transformed to metallic form with 1T or 1T’ phases. The phase transition provides an effective strategy for further improving the performance of field-effect transistors (FET) because 1T phase shows much decreased contact resistance at the electrodes of the transistors based on ultrathin MoS_2_ channels with 2H phase (Kappera et al. 2014; Li et al. 2021). Group 6 TMDs with 1T’ phase suggest a topological FET made of van der Waals heterostructures (Qian et al. 2014). Weyl Semimetallic and superconducting behavior supply a chance to study the fundamental correlation between new quantum phenomena and stacking structure (Cho et al. 2017; Kang et al. 2022).

In general, monolayer TMDs with MX_2_ exhibit 1H, 1T, and 1T’ phase as shown in Fig. 1b. Group 6 TMDs exhibit 1H and 1T’ phases. The 1T structure, which forms a rhombohedral (ABC) stacking, is known to be unstable. Most of these studies were conducted using semiconducting 1H phase-forming Bernal (ABA) stacking. The 1T’ phase is known to be a 2D quantum spin Hall insulator with a relatively large band gap (~ 0.1 eV) (Qian et al. 2014; Keum et al. 2015). It shows a distorted 1T structure owing to rhombohedral (ABC) stacking.

Scanning tunneling microscopy and spectroscopy (STM/STS) are powerful tools for characterizing the atomic and electronic structures of GBs. This review provides an overview of the recent progress made in the role of GBs in Group 6 TMDs in terms of their atomic and electronic properties analyzed using STM and STS measurements.

## Main text

### Atomic and electronic structure of tilt GBs in hexagonal phase

**Fig. 2:**
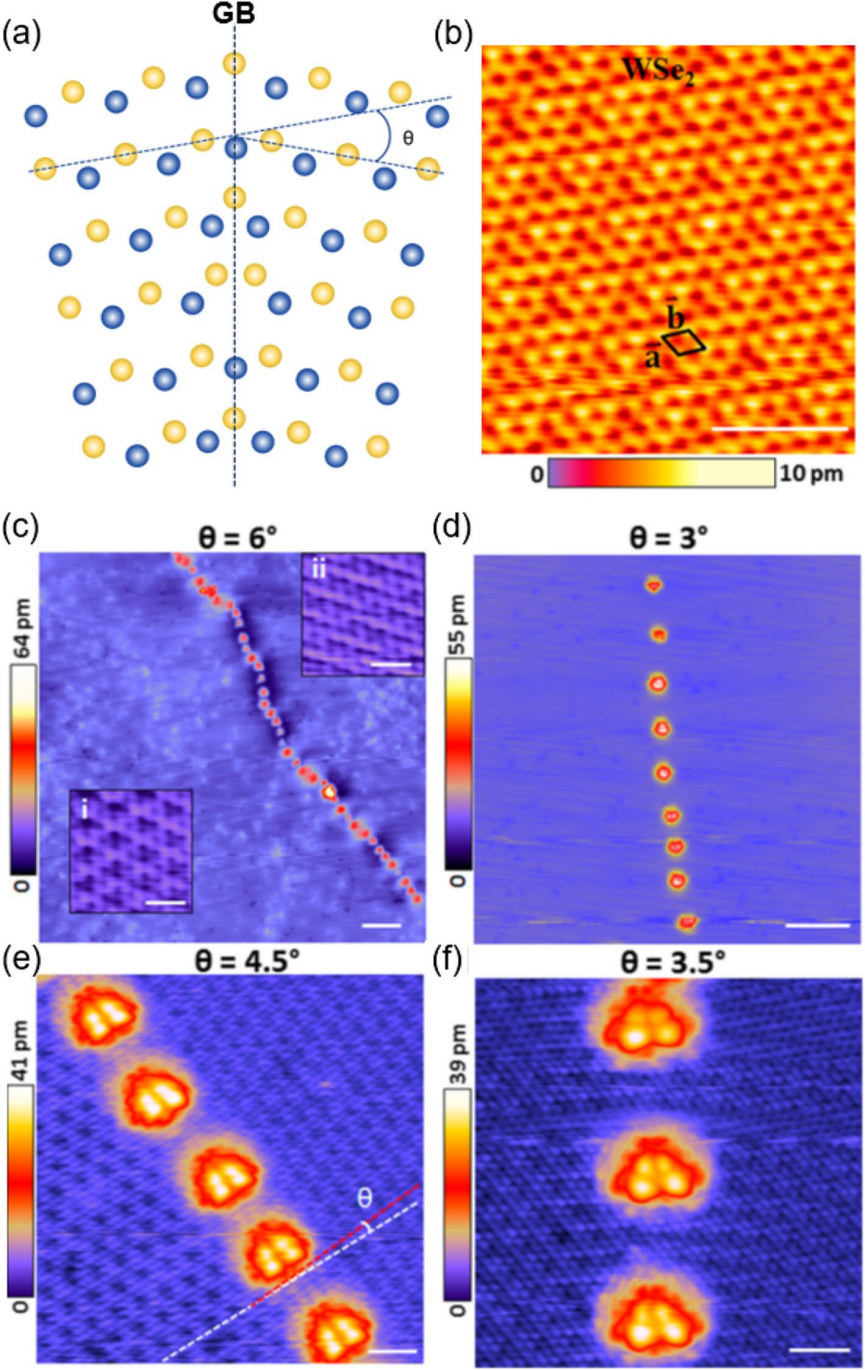
**a** Schematic of a GB with a misorientation angle (θ). **b** STM image of WSe_2_ transferred onto a graphite substrate. **c-f** STM images of GBs with various misorientation angles (θ = 6° to 3°) in WSe_2_. Scale bars are in (c, d) 10 nm, inset in (c), 1 nm, (e.f) 2 nm. **b-f** Reprinted with permission from Huang et al. (2016) (Nano Lett. 16, 3682–3688). Copyright (2016) American Chemical Society

GBs can be roughly classified into two categories: one is formed by facing two randomly distributed grains at a misorientation angle (θ) with each other, i.e., θ > 0°; the other is created with no misorientation angle between the grains, i.e., θ = 0°. The GBs with θ = 60, 120, and 180° are shown in Fig. 2a. The GBs with θ can further be classified as low-angle (0 < θ < 15°) and high-angle (θ > 15°) GBs. Such GBs are commonly observed in semiconducting phases (1H) and are mostly composed of dislocation cores, such as local 4–4, 5–7, 6–8, and 4–6 rings.

The surface of the hexagonal group 6 TMDs naturally reveals a hexagonal lattice in atomically resolved STM images, as shown in the 1H-WSe_2_ surface in Fig. 2b (Huang et al. 2016). When 1H–WSe_2_ was transferred onto a graphite substrate, low-angle GBs with θ = 3–6° were formed, which showed bright protrusions owing to the dislocation cores under negative tip bias voltages (Fig. 2c–f). In this case, the protrusions disappeared at positive bias voltages. Despite the dependence of the applied bias voltages on the local density of states (LDOS), protrusions are frequently observed in STM images corresponding to various GBs present in MoS_2_ and MoSe_2_ (Huang et al. 2016; Liu et al. 2016; Wang et al. 2018; Koós et al. 2019). The bias voltage-dependent protrusions are associated with the redistribution of the LDOS at the dislocation cores, eventually causing degradation of mobility and changes in transport characteristics. The variations depend significantly on the type of dislocation core, which is determined by the misorientation angle and materials. GBs with θ of 3.5° and 4.5° in 1H–WSe_2_ transferred onto graphite produce mid-gap states, which arise from the tetragonal dislocation core and surrounding strained hexagonal rings (Huang et al. 2016). According to the Burgers model, the interspacing between the protrusions increased as the misorientation angle decreased from 6° to 3°. The bandgaps depend on the tilt angle, underlying substrate and so on. In the case of 1H-MoS_2_ grown on highly ordered pyrolytic graphite (HOPG), a high-angle GB with θ = 25° is formed, which generates the defect states at the protrusions, even though these are below the conductance-band minimum, and causes the bandgap to decrease from 2.4 to 2.0 eV (Wang et al. 2018). However, in the GB with θ = 16° the bandgap increases to 4.4 eV, which is quite larger than that of 2.7 eV for the intrinsic 1H-MoS_2_ grown on HOPG. The bandgap changes at different positions of the GB. The bandgap measured at a dislocation core is smaller than that between two neighboring dislocation cores. Owing to the GB with θ = 18°, the bandgap of the 1H–MoS_2_ monolayer grown on graphite decreases from 2.40 ± 0.05 eV to 1.55 ± 0.05 eV, but gap states are not observed (Huang et al. 2016). The bandgaps of 1H − MoSe_2_ grown on graphite also decrease owing to GBs with θ = 5° and 22°(Koós et al. 2019).

1H − MoS_2_ having GBs with no misorientation, i.e., 0° and 60°, were grown on hBN and graphite through the interaction between MoS_2_ and substrates during the chemical vapor deposition process (Nakanishi et al. 2019). The crystalline structures of the substrate and three-fold (hBN) and six-fold (graphite) rotation symmetries limit the crystal orientations of the grown MoS_2_ islands, leading to the formation of only two orientations: 0° and 60°; the former is due to two well-stitched grains. The d*I*/d*V* spectrum for the GB with θ = 0° does not show any gap state, but it reveals a reduced bandgap from 2.2 to 1.8 eV by the strain of 2%. For the GB with θ = 60°, the boundary states exist at − 0.78 eV, exhibiting a reduced bandgap from 2.3 to 1.9 eV. Other cases without misorientation are discussed in the following section.

### Atomic and electronic structure of mirror twin GBs in hexagonal phase

Mirror twin grain boundaries (MTBs), which are representative cases of GBs with no misorientation, are frequently observed in Group 6 TMDs. This is related to their three-fold symmetry in the hexagonal lattice. MTBs are formed by facing two grains with mirrored symmetry and can be described as having a 60° rotation symmetry. A periodic MTB reveals metallic states within the bulk gap of 2D Group 6 TMDs with hexagonal phase. These metallic states in MTB along the FET channel may result in a leakage current (Yang et al. 2023). However, MTB networks provide opportunities for metallic contacts of the transistors with 2H phase (Najmaei et al. 2014; Diaz et al. 2016).

**Fig. 3:**
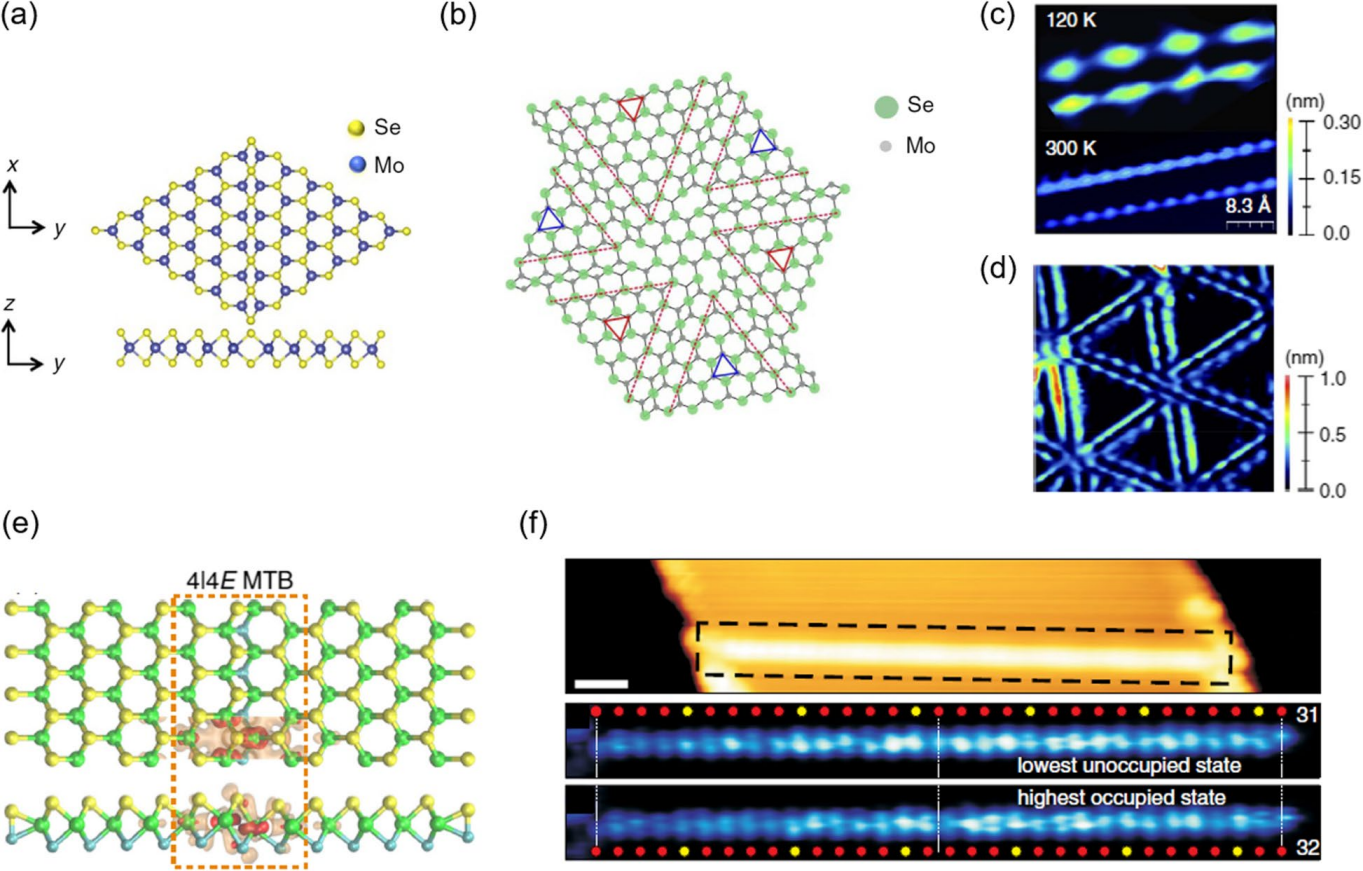
**a** Model structure of MTBs in MoSe_2_. **b** Arrangements of the three equivalent MTB directions gives rise to a cross-hatched grain boundary network. **c** STM image of MTB at a low temperature (120 K), exhibiting three times the periodicity than the atomic corrugation imaged at room temperature. **d** Large-scale low temperature STM image. **e** Top and side views of a ball-and-stick model of 4ǀ4E MTB in MoS_2_. **f** STM image of a 4ǀ4E MTB. STS maps of the dashed box area in the STM image at the energies of the highest occupied and lowest unoccupied states. **a-d** Reprinted with permission from Ma et al. (2017a, b) (Nature Commun. 8, 14231) CC BY 4.0. **e–f** Reprinted with permission from Jolie et al. (2019) (Phys. Rev. X 9, 011055) CC BY 4.0

Figure 3a shows the most commonly observed model structure of the MTBs in MoSe_2_, which has the lowest formation energy among the six possible MTBs (Lin et al. 2015; Batzill 2018). In an MTB, the two half-lattices of MX_2_ are terminated by chalcogen atoms, and the transition metals are rich. MTBs were first observed in 1999 in MoSe_2_ grown on MoS_2_ using STM (Murata et al. 1999). MTBs in the STM images appear as a hexagonal wagon-wheel-like pattern (Fig. 3d). However, these were not recognized as MTBs, but as Moiré patterns arising from a lattice mismatch of MoSe_2_ and the underlying MoS_2_ owing to the quantum confinement effect (Murata et al. 1999). In fact, in many cases it is not easy to identify their exact atomic structure only by STM images because STM images possess not only the geometrical structures but also the electronic properties. Therefore, STM measurements combined with other tools like transmission electron microscopy (TEM) and atomic force microscopy (AFM) can be helpful to explore the exact atomic structure. Indeed, Liu et al. (2014) reanalyzed the MTBs formed in MoSe_2_ on HOPG using STM combined with a high-resolution TEM. Furthermore, Barja et al. (2016) identified their precise atomic structure using STM images combined with non-contact AFM using CO-functionalized tips.

The bright contrast of the MTBs in STM images is strongly correlated with their electronic properties. The MTBs in MX_2_ exhibit metallic characteristics that are distinct from their semiconducting bulk properties. Considerable effort has been devoted to exploring the origins of MTBs (Batzill 2018 and references therein). Two fundamental principles in physics, namely, the charge density wave (CDW) transition and Tomonaga-Luttinger liquid (TLL) behavior, are acceptable to explain the metallic behavior in MTBs; however, the origin of the observed gap in the d*I*/d*V* spectra measured for MTBs is still controversial. A CDW is a prototypical case of Peierls instability, in which an equally spaced 1D chain is unstable (Grüner 1988). Indeed, periodic modulation with the tripling of the atomic lattice along the MTBs in 2H-MoSe_2_ manifests in the STM images obtained at 4 and 120 K, but they did not appear at room temperature (Fig. 3c) (Barja et al. 2016; Ma et al. 2017a, b). A bandgap of ~ 100 mV obtained at the Fermi level in the d*I/*d*V* spectra measured at 4 K also supports the theory of CDW transition. However, the measured gap is inversely proportional to the length of the GB, which cannot be explained by CDW transition alone (Xia et al. 2020). The gap dependence on the length of GB is explained by the quantum confinement effect, which is the spin-charge separation predicted by the finite-length TLL theory as MTBs mostly have a finite length. Recent experimental evidence obtained through gate-tunable MTB devices combined with STM and STS measurements further supports the idea that MTBs in 2H-MoSe_2_ exhibit a TLL ground state (Zhu et al. 2022). Angle-resolved photoemission spectroscopy spectra also support a TTL behavior, which is a pow law suppuration of the DOS (Ma et al. 2017a, b). However, despite of the evidences of TTL ground state, temperature dependent 4-point resistance measurements on 2H–-MoSe_2_ on 2H–MoS_2_ substrate show two changes in resistance at ~ 235 K and ~ 205 K. Such resistance increases are commonly discovered in Peierls systems and CDW transitions (Ma et al. 2017a, b). Therefore, it is still quite difficult to determine the origin of MTBs.

The debate on the underlying mechanism of charge modulation in MTBs is ongoing even for 2H–MoTe_2_ and 2H–MoS_2_. The periodic modulation with the tripling of the atomic lattice and the energy gap in the MTBs in 2H–MoTe_2_ were interpreted as a 1D Peierls-type CDW transition at 77 K (Zhu et al. 2017; Dong et al. 2018; Wang et al. 2020). In 2H–MoS_2_, two types of MTBs were identified: one exhibited two parallel lines in the d*I*/d*V* map, similar to the type described above, whereas the other exhibited a single line (Ma et al. 2017a, b; Jolie et al. 2019). The periodicity of the latter is a doubling of the atomic lattice, and the MTB exhibits the 4ǀ4E structure, as shown in Fig. 3e (Murray et al. 2020). In this MTB, instead of a CDW transition, evidence of spin-charge separation was found.

MTBs were not observed in WSe_2_ because of their high formation energies. However, recently, it was reported that V-doping, which is carried out by the co-deposition of V and W during the molecular beam epitaxy growth of WSe_2_, can result in MTB formation in WSe_2_ (Pathirage et al. 2023). The electronic properties, the origin of MTBs in WSe_2_ and other WX_2_, and substrate effects on the electronic properties are not mentioned in this review as they remain unclear.

### Topological grain boundary states in distorted trigonal phase

Monolayer TMDs with 1T’–MX_2_ are widely known as quantum spin Hall insulators whose edges are topologically protected from backscattering owing to time-reversal symmetry and spin–orbit coupling (Qian et al. 2014; Keum et al. 2015). Topological states are found at the edges or interfaces between the 1T’ phase and insulating or semiconducting materials, such as the 1H phase (Fei et al. 2017; Tang et al. 2017; Jia et al. 2017; Ugeda et al. 2018; Yan et al. 2020). In addition to this, nonsymmorphic symmetry (screw rotation and glide-reflection symmetries) in a GB can also create topological states (Ran et al. 2009; Juričić et al. 2012; Slager et al. 2014; Kim et al. 2020). This section focuses on the symmetry-enforced topological states and their atomic structures.

**Fig. 4:**
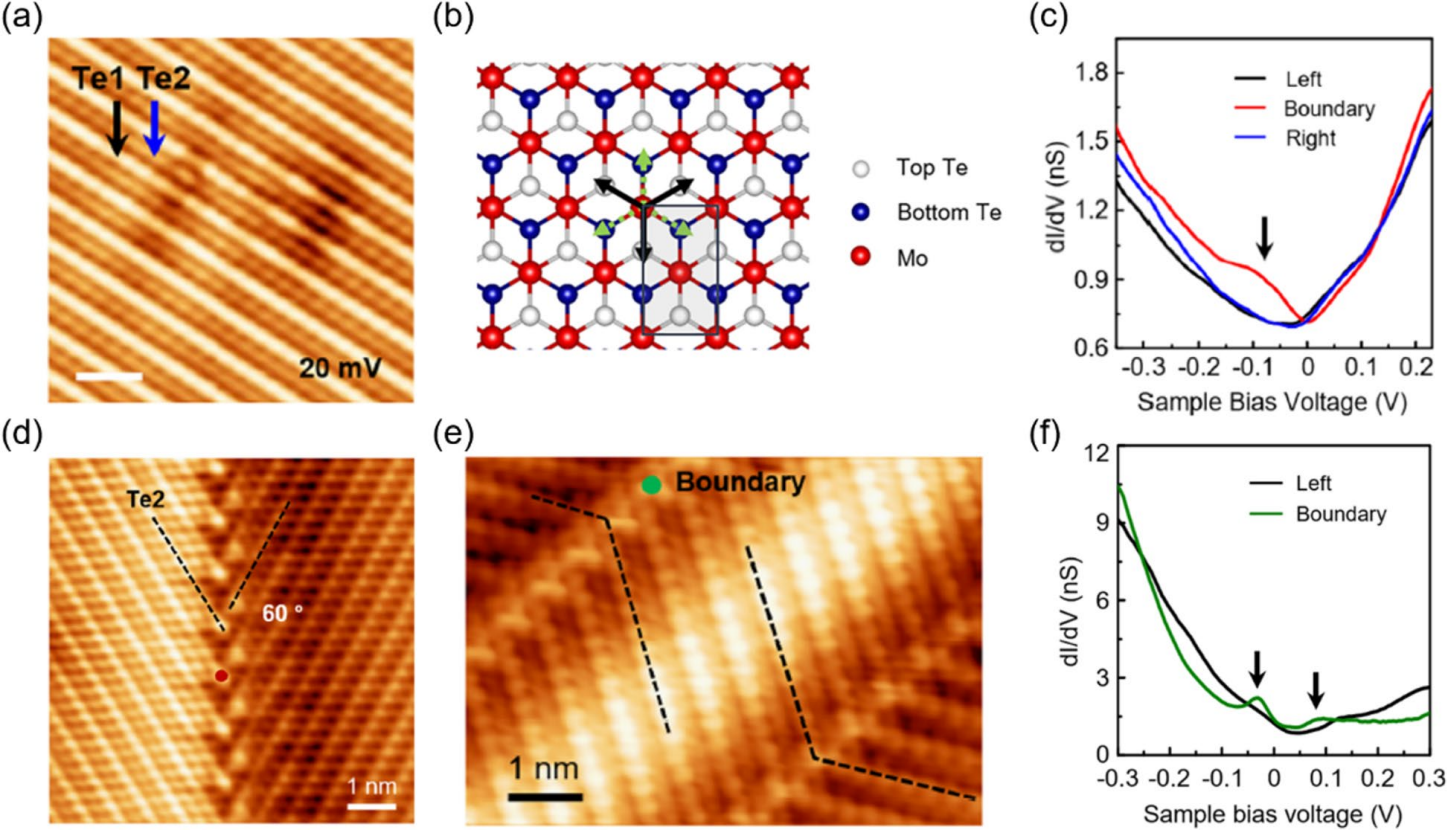
**a** STM image of the 1T′-MoTe_2_ surface. **b** Three symmetry-equivalent directions (black arrows) of structural distortion in the 1T structure and their three opposite directions (green dotted arrows). **c** Averaged d*I*/d*V* spectra obtained at left area (black line) and right area (blue line) and d*I*/d*V* spectrum obtained at the position indicated by the red dot in (d) (red line). **d-e** STM images of 60° glide-reflection and 120° two-fold rotation boundaries, respectively. **f** Averaged d*I*/d*V* spectrum obtained at left area (black line) and d*I*/d*V* spectrum obtained at the position indicated by the green dot in (**e**) (green line). Reprinted with permission from Kim et al., (2020), (Nano Lett. 20, 5837 − 5843). Copyright (2020) American Chemical Society

The surface of 1T’-MTe_2_ has revealed that the chalcogen atoms form quasi-one-dimensional parallel chains with height variations because of the distorted trigonal structure, as shown in Fig. 4a. In the STM image of 1T’–MoTe_2_, two Te atom lines were clearly identified, and these alternating lines facilitate the recognition of the symmetry of the GB (Kim et al. 2020; S–H Kang et al. 2022). It was theoretically predicted that three orientation variants, resulting from the three equivalent directions of structural distortion in the parental 1T phase, can produce GBs with 120° rotational symmetry (Li et al. 2016). Indeed, GBs with an angle of 120°in 1T’-WTe_2_ were formed using the thermal expansion coefficients between WTe_2_ and indium substrate (Wang et al. 2019). GBs were created and manipulated in 1T’–WTe_2_ using STM tip pulses (Pedramrazi et al. 2019). In addition to the variants, it was reported that a structural phase transition from 1 to 1T′ through dimerization of adjacent Mo rows can produce six orientation variants, resulting from the sixfold improper rotational symmetry (black and gray dotted arrows in Fig. 4b) (Kim et al. 2020). This phase transition can create four types of GBs with 60° mirror, 60° glide reflection, 120° two-fold rotation, and 120° screw rotation symmetries. Among these, the 60° glide reflection and 120° two-fold rotation boundaries were experimentally observed in 1T’–MoTe_2_.

The STM image in Fig. 4d clearly reveals the 60° glide-reflection symmetry that consists of reflection and translation by half of the period with respect to the GB. The d*I*/d*V* spectrum measured at the GB has a peak at around − 70 mV (Fig. 4c), which is localized at the GB within a width of ~ 2 nm. This peak is known as the Weyl semimetallic state produced by band crossing owing to nonsymmorphic symmetry (Young et al. 2015). The GB with 120° exhibits two-fold rotation symmetry (Fig. 4e) and does not possess any topological state (Fig. 4f). In the d*I*/d*V* spectrum, two peaks originate from the gapped states; the intensities of the peaks in the d*I*/d*V* maps are much weaker than that of the peak shown in the GB with 60°. This observation was also supported by density functional calculations. As GBs are formed using strain and tip pulses (Wang et al. 2019; Pedramrazi et al. 2019), it is expected that manipulation between GBs with and without a topological state can be realized in the near future.

### Potential applications and perspectives

Various GBs and their intriguing properties in 2D TMDs have been remarkably boosted for GB-based functional devices. GB-based devices with hexagonal phase can be developed to non-volatile resistive random-access memory and neuromorphic devices. In 2015, a GB in monolayer MoS_2_ have been demonstrated for three-terminal memristors (Sangwan et al. 2015). The memristive behavior depends on the orientations of GBs (called intersecting, bisecting and bridge memristors) which provide versatile functionalities. In 2018, a multi-terminal hybrid memristor and transistor has been experimentally realized using polycrystalline monolayer MoS_2_ (Sangwan et al. 2018). MoSe_2_ with MTBs networks has a potential for the applications of electrocatalysis like hydrogen evolution reaction catalysis, because the metallic states in MTBs are known as catalytically active sites comparing to the semiconducting regions of 1H- phase (Jaramillo et al. 2007; Kosmala et al. 2018). Furthermore, selective metal decoration on MTB networks can be possible and the metal deposited area can be used for metallic contacts of 2D TMD-based FET (Y Ma et al. 2017a, b). Topological states enforced by nonsymmorphic symmetry of GBs in group 6 TMDs with 1T’ phase possesses a great potential to use a topological FET. The creations and manipulations of such topological states which can be induced by strain may provide new opportunities for future research and development of new device applications.

## Conclusions

This review briefly discussed the atomic and electronic properties of various GBs in 1H− and 1T’−MX_2_, which were investigated using STM and STS measurements. Most of the GBs formed in 1H−MX_2_ with a misorientation angle (θ > 0°) produced gap states (with a few exceptions) and reduced the bandgap. GBs with no misorientation (θ = 0°) formed in 1H−MoS_2_ grown on graphite did not produce gap states, but they reduced the bandgap. MTBs were frequently observed when transition metals were abundant (two parallel lines in the STM images) or deficient (a single line in the STM image). The origins of MTBs were intensively studied, and the CDW transitions and TTL behaviors were considered acceptable for explaining the metallic properties of MTBs. In 1T’−MX_2_, topological states were found in the 60° nonsymmorpic GB, which did not exist in the 120° symmorphic GB. This short review is expected to improve our understanding of the versatile properties of GBs formed in 2D Group 6 TMDs.

## Data Availability

Not applicable.

## Abbreviations

2D: Two dimensional
AFM: Atomic force microscopy
CDW: Charge density wave
FET: Field-effect Transistor
GB: Grain boundary
LDOS: Local density of states
MTB: Mirror twin grain boundary
STM: Scanning tunneling microscopy
STS: Scanning tunneling spectroscopy
TEM: Transmission electron microscopy
TMD: Transition metal dichalcogenide
TTL: Tomonaga-luttinger liquid
